# Biocompatible ι-carrageenan-γ-maghemite nanocomposite for biomedical applications – synthesis, characterization and *in vitro* anticancer efficacy

**DOI:** 10.1186/s12951-015-0079-3

**Published:** 2015-03-03

**Authors:** Maya Raman, Viswambari Devi, Mukesh Doble

**Affiliations:** Bioengineering and Drug design Lab, Department of Biotechnology, IIT-Madras, Chennai, 600036 India

**Keywords:** ι-carrageenan, γ-maghemite nanoparticles, Nanocomposite, Biocompatible, Apoptosis, Drug delivery, Hyperthermia

## Abstract

**Background:**

Carrageenans are naturally occurring hydrophilic, polyanionic polysaccharide bioploymers with wide application in pharmaceutical industries for controlled drug delivery. Magnetic nanoparticles with their exceptional properties enable them to be an ideal candidate for the production of functional nanostructures, thus facilitating them for biomedical applications. The development of novel nanocomposite by coupling the synergistic effects of the sulfated polysaccharide (iota carrageenan) and a magnetic nanoparticle (maghemite) may offer new interesting applications in drug delivery and cancer therapy. The nanocomposite was characterized by ultraviolet–visible spectroscopy, high resolution scanning electron microscopy, dynamic light scattering analysis, Fourier transform infrared spectroscopy and powder XRD to highlight the possible interaction between the two components. Biocompatibility and the anticancer efficacy of the nanocomposite were assayed and analysed *in vitro*.

**Results:**

Results suggested that iota carrageenans have electrostatically entrapped the maghemite nanoparticles in their sulfate groups. Biocompatibility of the nanocomposite (at different concentrations) against normal cell lines (HEK-293 and L6) was confirmed by MTT assay. Hoechst 33342 and 7-AAD staining studies under fluorescent microscopy revealed that the nanocomposite is able to induce appoptosis as the mode of cell death in human colon cancer cell line (HCT116). Cell apoptosis here is induced by following the ROS-mediated mitochondrial pathway, combined with downregulation of the expression levels of mRNA of XIAP and PARP-1 and upregulation of caspase3, Bcl-2 and Bcl-xL.

**Conclusions:**

This novel nanocomposite is biocompatible with potential properties to serve in magnet aided targeted drug delivery and cancer therapy.

**Electronic supplementary material:**

The online version of this article (doi:10.1186/s12951-015-0079-3) contains supplementary material, which is available to authorized users.

## Introduction

The advancements in the area of nanoparticles and nanotechnology have offered an understanding and management of the materials at atomic and molecular levels. It has also assisted in fabricating advanced materials with added magnetic, electrical, optical and biological properties for pharmaceutical and biomedical applications [1]. Nanovectors in the field of delivery are promising novel tools for controlled release of drugs. In recent years, the unique novel properties (superparamagnetism, high coercivity, low Curie temperature and high magnetic susceptibility) of iron oxide nanoparticles (magnetite, maghemite) have been exploited to make it inevitable in magnetic resonance imaging (MRI), magnetic fluid hyperthermia, controlled drug delivery systems and cancer therapy [2]. Nevertheless, these magnetic nanoparticles are functionally efficient to perform these tasks only when incorporated with suitable polymer [3,4]. Encapsulating magnetic nanoparticles within a polymer not only stabilizes the nanoparticles but also provides various chemical functionalizations. Many polysaccharide-based magnetic nanocomposites such as, magnetite (Fe_3_O_4_)-dextran, Fe_3_O_4_-chitosan, Fe_3_O_4_-alginate, Fe_3_O_4_-heparin, Fe_3_O_4_-pullulan acetate, Fe_3_O_4_-starch, Fe_3_O_4_-κ-carrageenan, maghemite (γ-Fe_2_O_3_)-dextan/sucrose, were successfully used in bioseparation and purification [5,6], bioassays and sensors [7-9], biolabelling and imaging [10,11], cancer hyperthermia [12,13], cardiovascular therapies [14] and drug delivery [15,16].

Carrageenans are naturally occurring high molecular weight, hydrophilic polysaccharides extracted from red sea weeds of phylum Rhodophyta. These are polyanionic linear sulfated galactans with a sequence of D-galactopyranose and 3,6-anhydrogalactopyranose residues bonded by alternating α(1→3) and β(1→4) linkages [17]. Based on the number and position of ester sulfate groups (−SO_3_^−^) on the galactose units, these are classified into kappa (κ) -, iota (ι) -, and lambda (λ) – carrageenan (main commercial variants). ι *–*Carrageenan (ι *–*car) is composed of D-galactose-4-sulphate (G4S) and 3, 6-anhydro-D-galactose-2-sulfate (DA2S). These biocompatible and biodegradable biomacromolecules are extensively used in food and pharmaceutical industries. In pharmaceutical industry, these play a significant role as gelling agents in controlled drug release and prolonged retention. Their anticancer, antioxidant, anticoagulant, antihyperlipid, antiviral and immunomodulatory activities have gained several pharmacological applications [18,19]. The ι-carrageenan has been reported to possess high metal binding activity [20]. They are reported to act as sorbents and aid in binding heavy metals including yttrium (Y^3+^) and lead ions (Pb^2+^) [20]. This intrinsic metal binding property of carrageenans and other polysaccharides are successfully employed in nanoparticle synthesis and encapsulation; and hence, in making nanoparticles suitable for a broad spectrum of biomedical and biotechnological applications [21].

Carneiro et al. [22] reported that γ- Fe_2_O_3_ nanoparticles coated citrate and rhodium (II) citrate enhance cytotoxicity on breast carcinoma. Degraded ι – car was also reported to have antitumor activity towards human osteosarcoma cell line both *in vitro* and *in vivo* [23]. Hence, the synergic effect of the nanoparticles and the polysaccharides could be a new area of research which could confer beneficial functionalities and multiple bio-applications to the product developed. In our study, γ – maghemite (Fe_2_O_3_) nanoparticles were combined with ι – car in a significant way to develop a novel nanocomposite material. These were then characterized and subjected to *in vitro* studies to open up their possible range of applications in cancer research.

## Results and discussion

**Figure 1:**
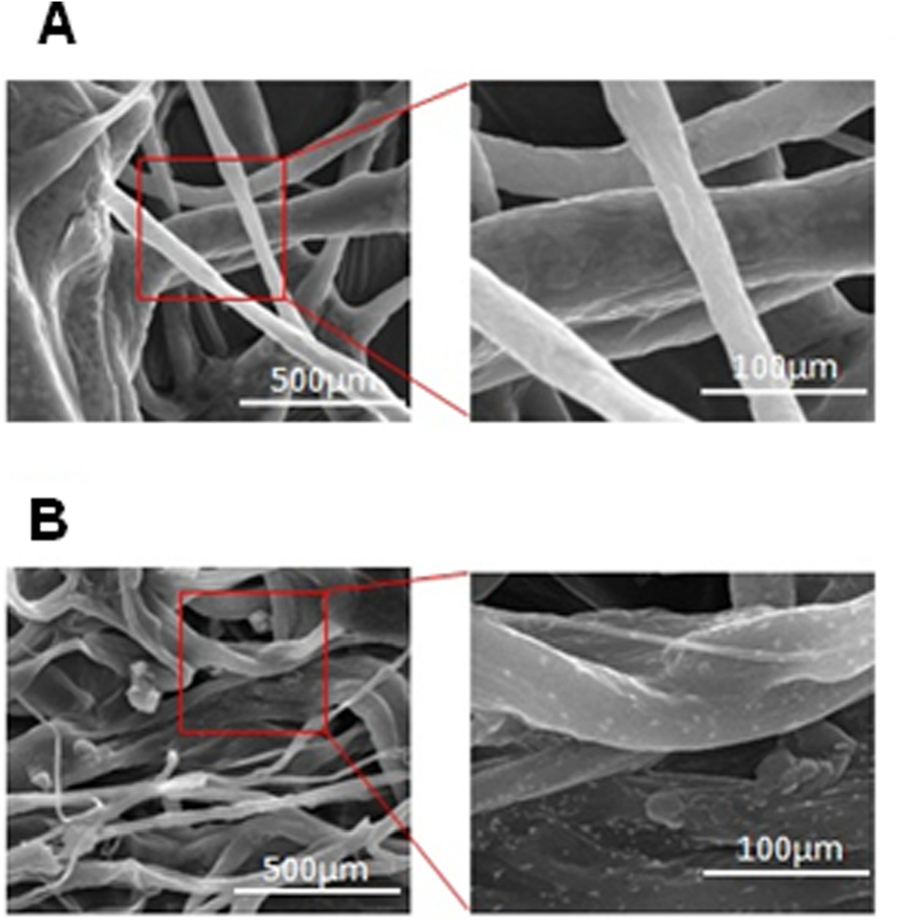
**SEM micreograph (A) carrageenan with no nanoparticles (B) carrageenan with maghemite nanoparticles forming** **ι** **-car-** **γ** **-Fe**_**2**_ **O**_**3**_ **nanocomposite.**

ι-car is an anionic polysaccharide with high ζ-potential value. This anionic nature is because of the sulfate group in each unit of D-galactopyranose-4-sulfate and 3, 6-anhydrogalactose units. The ζ-potential of ι-car was −52.84 ± 3.6 mV and that of ι-car-γ-Fe_2_O_3_ nanocomposite was −7.70 ± 2.8 mV. This lowering in surface charge of the latter could be attributed to the inclusion of the positively charged γ- Fe_2_O_3_ nanoparticles (+33 ± 3 mV) to the surface of anionic ι-car. The electrostatic attraction between anionic sulfate groups (−SO^−^_4_) on the carrageenan molecule and cationic patches (−Fe^2+^) on maghemite may interact and contribute to the nanocomposite size and zeta potential [24]. Similar results were reported in many studies and this change in the ζ-potential could be highly dependent on the concentration of components in the composite [25,26]. SEM micrographs showed maghemite nanoparticles dispersed throughout the carrageenan microfibrils (Figure 1). However, for *in vitro* studies, the nanoparticle dispersed microfibrillar composite was ultrasonicated and washed in buffer to get nanometer sized particles that were larger than maghemite nanoparticles (21 ± 3.6 nm) used in the preparation of the composite. The average particle size ranging from 200 – 550 ± 10 nm was observed and this wide range of distribution may be due to their aggregation (Additional file 1).

The UV absorption maxima for ι-car was observed at 296 nm, which shifted to 304 nm in the nanocomposite possibly indicating structural modification in ι-car, which might be due to the entrapment of maghemite nanoparticles [27] (Additional file 2).

**Figure 2:**
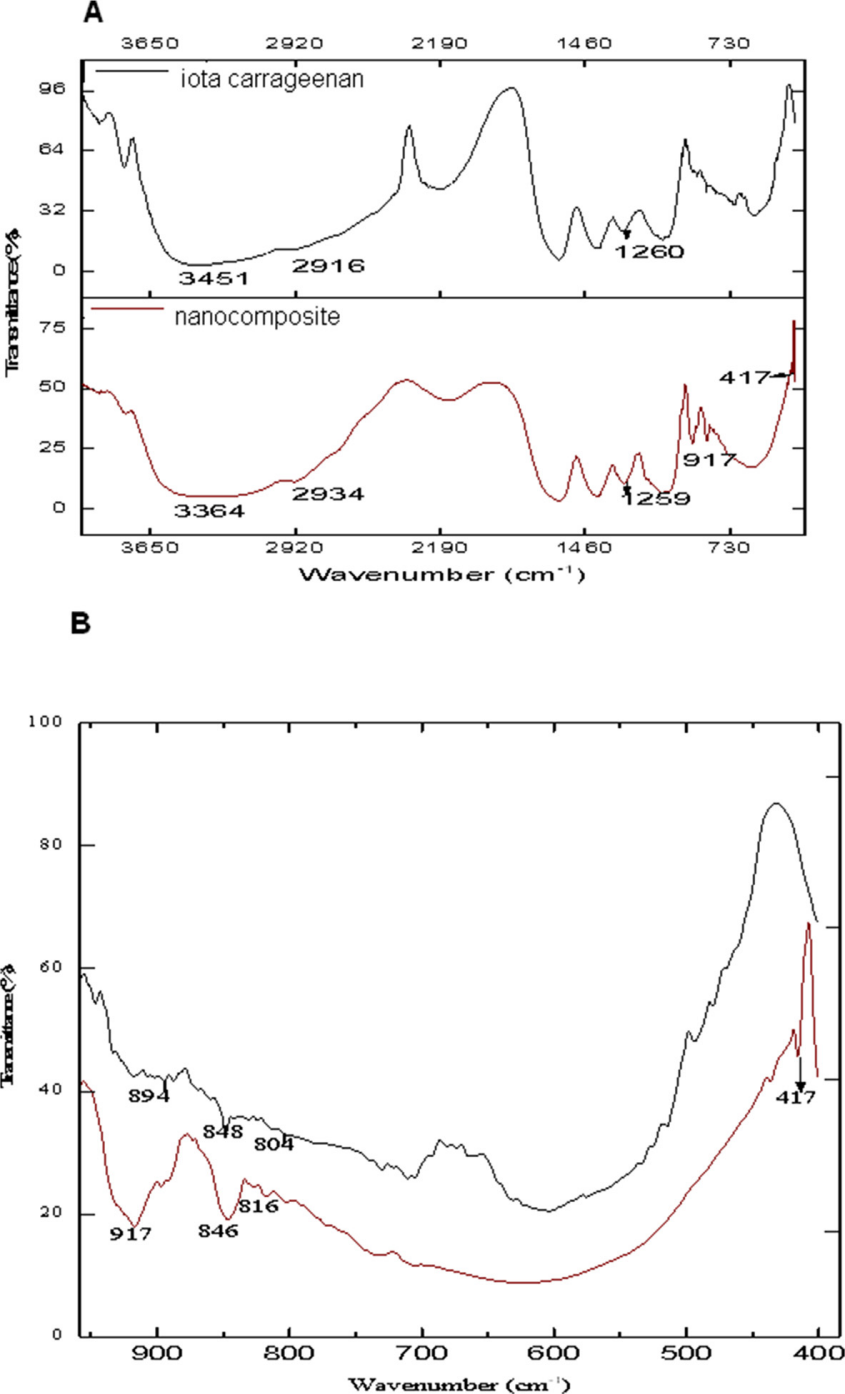
**FTIR spectrum of (A)** **ι** **-car and** **ι** **-car-** **γ** **-Fe**_**2**_ **O**_**3**_ **nanocomposite (B) magnified lower finger print region of FTIR spectrum of** **ι** **-car and** **ι** **-car-** **γ** **-Fe**_**2**_ **O**_**3**_ **nanocomposite.**

The FTIR bands specific to ι-car are observed in both the samples (Figure 2, Table 1), with few exceptions in the lower fingerprint region (800–400 cm^−1^). Broad bands are observed between 3400–3000 cm^−1^ corresponding to the hydroxyl groups in the polysaccharide which is responsible for the hydrophilic nature of the carrageenan [28]. The bands between 2900–2700 cm^−1^ are assigned to the asymmetrical stretching vibrations in -CH_2_ of the galactose units [28]. The characteristic band in the 1210–1260 cm^−1^ region was attributed to the sulfate esters that were present in both, confirming the retention of the sulfation in the latter [29]. The peak at 1070 cm^−1^ is attributed to glycosidic linkages in the polysaccharides [29]. Presence of 3, 6-anhydro-D-galactopyranose units in both was confirmed from the presence of bands at 894 and 917 cm^−1^, and that of D-galactopyranose-4-sulfate (G4S) units by the presence of bands at 848 and 846 cm^−1^. The band specific to ι-car appears at 805 cm^−1^,which indicates the presence of sulfate group at C2-position in the 3, 6-anhydrogalactose unit (DA2S) [30]. This band however shifts to lower wavenumbers in the spectrum of the nanocomposite. This as well as the shift observed at 917 cm^−1^ in the nanocomposite may be due to the interaction of maghemite nanoparticles with the sulfate ester group in the 3, 6- anhydrogalactose-2-sulfate units. The appearance of sharp intense peak at 417 cm^−1^ corresponds to Fe-O stretch (Figure 2B) [31]. This could possibly be due to the impregnation of iron nanoparticles in ι-car mostly by electrostatic interaction with the sulfate groups of 3, 6-anhydrogalactose-2-sulfate units [27].

**Table 1:** **FTIR spectrum assignments**

**Functional groups**	**Wavelength (cm** ^**−1**^ **) of corresponding functional groups (** **ι** **-car)**		**ι** **-car-** **γ** **- Fe**_**2**_ **O**_**3**_ **composite**
Hydroxyl	3000 - 3600	3451	-3364
C-H stretch	2900 - 2700	2916	-2934
Ester sulfate	1220 - 1260	1260	-1259
3,6 anhydrogalactose	928 - 933	894-	917
G4S	840 - 850	848-	846
3,6 anhydrogalactose – 2- sulfate	800 - 805	804-	816
Fe –O stretch	500 - 400	---	417

**Figure 3:**
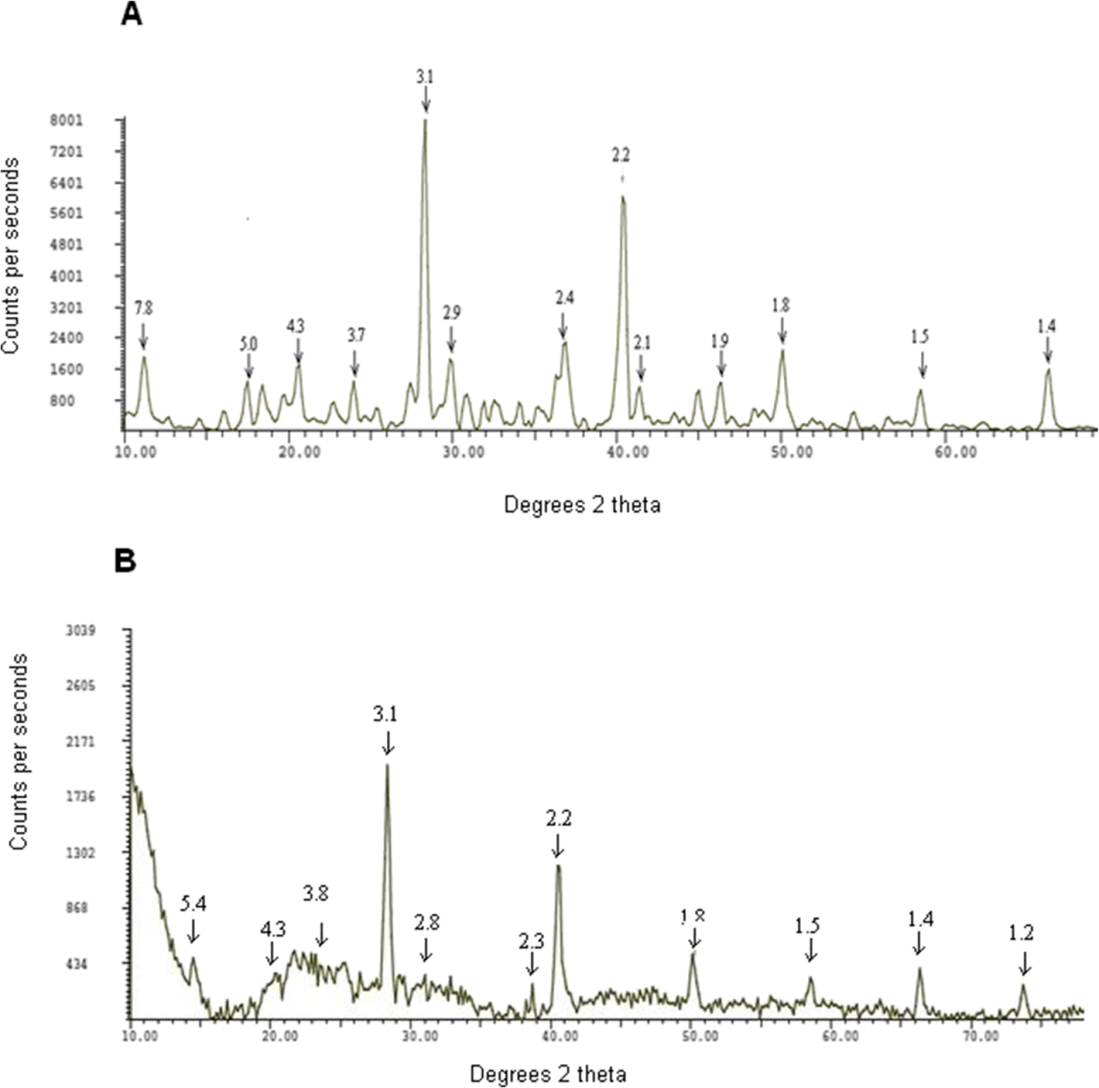
**Powder XRD diffractogram of (A)** **ι** **-car and (B)** **ι** **-car-** **γ** **-Fe**_**2**_ **O**_**3**_ **nanocomposite.**

X-ray powder diffraction pattern of ι-car and ι-car-γ-Fe_2_O_3_ nanocomposite indicate intense peaks at Bragg angles (2θ), 28°and 40°, while less intense peaks at 36°, 50°, 11°, 29°, 20°, 66°, 17°, 23°, 46°, 18°, 41°, 45° and 58°. γ-Fe_2_O_3_ nanoparticles have intense peaks at 35°, 63°, 57° and 30°. The XRD-diffractogram of the nanocomposite have the two intense peaks for ι-car (28° and 40°) and two peaks specific for γ-Fe_2_O_3_ nanoparticles (66° and 58°) and other characteristic peaks of its own at 14°, 25° and 26° (Figure 3, Table 2) [32,33]. Diffraction studies by Millane et al. [32], Janaswamy and Chandrasekaran [33] have shown that ι-car has a double helical structure with sulfate protruding away from the centre of helix. It has a trigonal lattice arrangement with small changes in the unit cell dimensions when it interacts with the nanoparticles.

**Table 2:** **Peak intensities of (A)** ι**-car and (B)** ι**-car-** γ**- Fe**_**2**_ **O**_**3**_ **composite from PXRD diffractogram**

**(A) PXRD diffractogram of** **ι** **-car**			**(B) PXRD diffractogram of** **ι** **-car-** **γ** **- Fe**_**2**_ **O**_**3**_ **composite**		
2θ	d spacing (A°)	I/100	2θ	d spacing (A°)	I/100
11.202	7.8924	24	14.507	6.1008	10
17.512	5.0602	16	20.517	4.3254	6
18.414	4.8144	14	23.221	3.8274	12
20.517	4.3253	20	28.33	3.1478	45
23.973	3.7091	16	31.034	2.8794	7
28.33	3.1478	100	32.837	2.7253	7
29.832	2.9926	23	38.696	2.325	6
36.743	2.444	27	40.499	2.2256	28
40.349	2.2335	75	50.265	1.8137	9
41.401	2.1792	14	58.528	1.5758	7
45.007	2.0126	13	66.341	1.4079	8
46.359	1.957	16	73.703	1.2844	6
50.115	1.8188	25			
58.528	1.5758	13			
66.341	1.4079	19			
73.553	1.2866	11			

**Figure 4:**
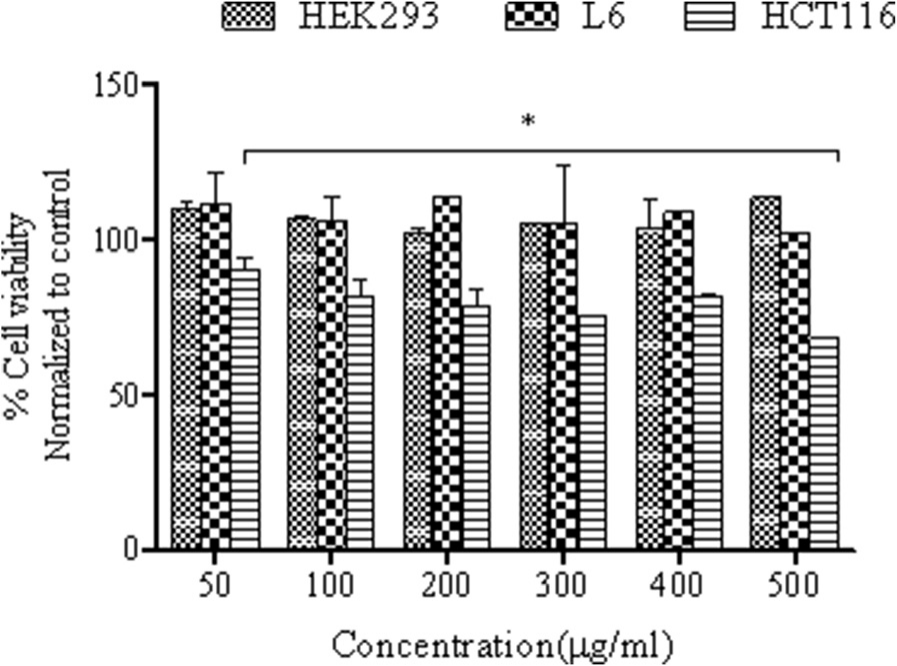
**Cell proliferation assay: viability of various cells (HEK293, L6 and HCT116) treated with** **ι** **-car,** **γ** **-Fe**_**2**_ **O**_**3**_ **and** **ι** **-car-** **γ** **-Fe**_**2**_ **O**_**3**_ **nanocomposite.** Significant difference (*, p < 0.01) is observed between normal cell lines (HEK293 and L6) and HCT116. Concentration dependent decrease in the viability is observed in HCT116 cell lines.

Cell proliferation assay for ι-car (1000 μg/ ml) using 3-[4, 5-dimethythiazol-2-yl]-3, 5-diphenyltetrazolium bromide dye showed, 75.4% viability of HCT116 cells and no cytotoxicity in HEK and L6 (more than 90% of viable cells). Supportingly, Arrifin et al. [34], have observed that iota carrageenan was non-cytotoxic to normal and cancer intestinal and liver cell lines even at 2000 μg/ ml. MTT assay with γ-Fe_2_O_3_ showed that the nanoparticles were cytotoxic to HEK, L6 and HCT116 cell lines at 50 μg/ ml and above (Additional file 3). This agrees with the studies of Prodan et al. [35], which have demonstrated that γ-Fe_2_O_3_ nanoparticles upto 30 μg/ ml concentration are non-cytotoxic on HeLa cells. The ι-car-γ-Fe_2_O_3_ nanocomposite treatment induced dose-dependent death of HCT-116 cells (reduction of cell viability from 98.8 to 68.4% with an increase in the concentration from 50–500 μg/mL, in 24 hours (p < 0.01)). The nanocomposite had no effects on the viability of HEK293 and L6 cell lines even at the highest concentrations tested (Figure 4). The concentrations of ι-car (700 μg/ ml) and γ-Fe_2_O_3_ (4 μg/ ml) in nanocomposite, when used independently had no effects on the viability of HEK293 and L6 cell lines and hence were biocompatible.

Nanocomposites comprising of maghemite have been recognized for their anticancer properties [36,37] and ι-car-γ-Fe_2_O_3_ nanocomposite is a novel compound that could be a potent inducer of apoptosis in various cancer cell lines. This could activate the extrinsic or intrinsic apoptotic pathways by altering the expression of apoptosis-associated or signaling proteins, cell cycle regulatory proteins and transcription factors. However, the molecular and cellular mechanism underlying these effects in HCT116 has not yet been investigated till date.

**Figure 5:**
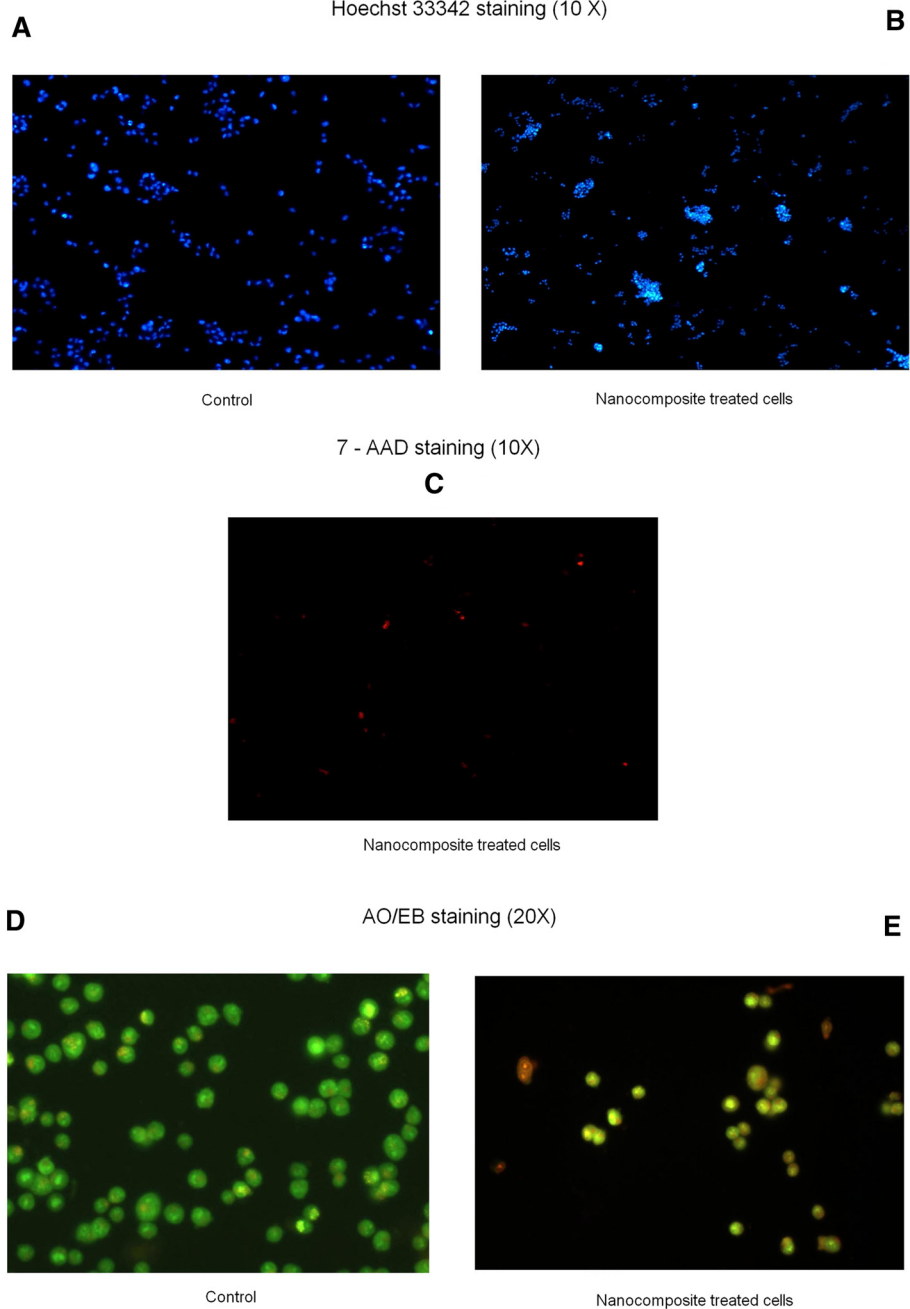
**Apoptosis studies: ι-car-γ- Fe**_**2**_ **O**_**3**_ **nanocomposite led to apoptotic characteristics in HCT116 cells; (A) Control-Hoechst 33342 staining (10X), (B) Nanocomposite treated cells-Hoechst 33342 staining (10X), (C) Nanocomposite treated cells-7-AAD staining (10X), (D) Control-AO/EB staining (20X) and (E) Nanocomposite treated cells- AO/EB staining (20X).**

The morphological changes in the HCT116 cells and its nucleus, induced by apoptosis were examined with different dyes. Apoptotic bodies (apoptosomes) were observed with Hoechst 33342 staining in nanocomposite-treated cells, but not in the control. These changes might include chromatin condensation, membrane blebbing and cell shrinkage. 7-AAD staining indicates compromised cellular membrane (late apoptotic and necrotic cells), while live cells with intact cell membranes remained dark (Figure 5A- C). Necrotic cell death might not be significant [38]. Figure 5 (D, E) shows the results of nanocomposite treated cells stained with acridine orange and ethidium bromide for 24 h. Number of viable cells here had decreased significantly. Apoptotic cells appear bright green or reddish with fragmented nuclei. The decrease in green fluorescence observed in treated cells when compared to control could be due to the reduction in the accessibility of nucleic acid by AO or reduced overall amount of DNA in the cells which undergo apoptosis. Cells which undergo apoptosis are permeable and, hence show increased fluorescence with EB. Nanocomposite-treated cells showed a significant reduction in the cell numbers and about 82% of cells were either orange or bright green apoptotic cells (apoptosomes) with fragmented and condensed nuclei [39,40]. In the control, the cells were healthy with no fragmented nuclei.

**Figure 6:**
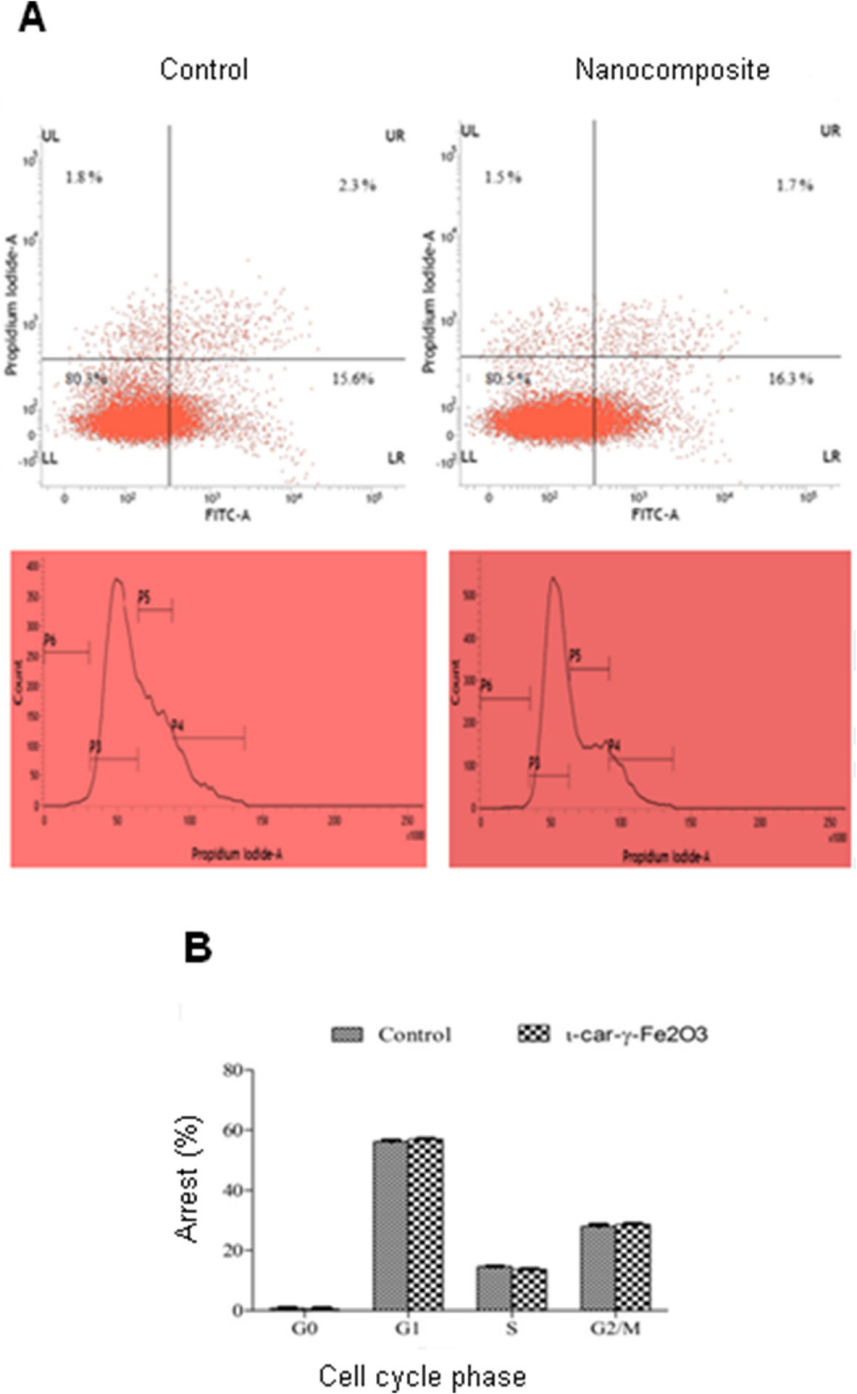
**Apoptosis studies: (A) Quantification of apoptosis by the annexin-V/PI double staining assay using flow cytometry; LL (low left), LR (low right), UR (upper right), and UL (upper left) denote viable (live), early apoptotic, late apoptotic and necrotic cells, respectively. (B)** The cell cycle analysis performed by flow cytometry showing percentage of arrest in different phases of cell cycle (P3-G1, P4-G2/M, P5-S and P6-G0 phases).

Annexin-V/PI double-staining and flow cytometry revealed that the nanocomposite effectively induced apoptosis in HCT-116 cells. The proportion of apoptotic cells (lower right quadrant) increased from 15.62% in untreated cells to 16.3% in nanocomposite-treated cells in 24 hours (Figure 6A). Compared to the ROS in the control, it is found that 1 mM of ascorbic acid markedly reduced the ROS level (59.7 ± 4.6% of control) in HCT116 cell lines. However, pre-incubation with ι-car-γ-Fe_2_O_3_ nanocomposite (500 μg/ ml) increased the ROS levels significantly (80.8 ± 0.4% of control, p < 0.01).

ROS are the byproducts of normal cellular oxidative process and are involved in the initiation of apoptotic and inflammation signaling. Increased ROS levels induce depolarization of the mitochondrial membrane which produces an increased level of pro-apoptotic molecules in the cells [41]. Oxidative stress indicates the imbalance between pro-oxidants and anti-oxidants and this is controlled by multiple factors, of which imbalances caused by cellular damage is a critical one. ROS play a key role in oxidative stress, and are generated as a by-product of cellular metabolism, primarily in the mitochondria [42]. The accumulation of ROS may lead to various forms of reversible and irreversible oxidative modifications to the cellular proteins, lipids and DNA, thus, accounting for cellular damage [43]. Depending on the extent of oxidative stress, elevated levels of ROS can induce proliferation, growth arrest, senescence and apoptosis [44].

**Figure 7:**
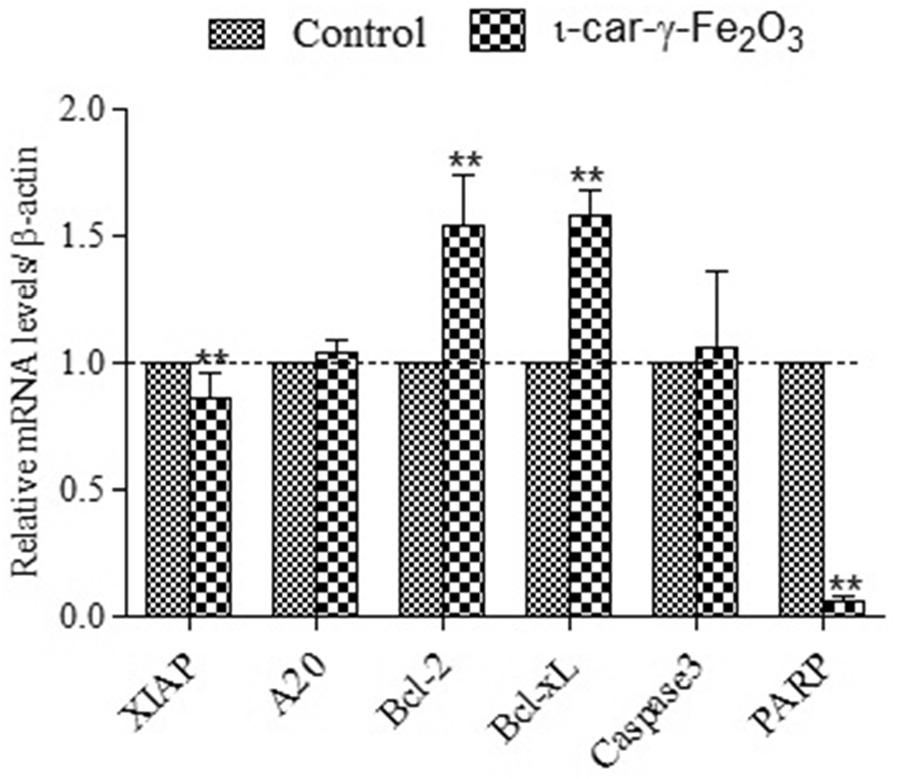
**Real-time polymerase chain reaction: Activity of X-linked inhibitor of apoptosis, A20, Bcl-2, Bcl-xL, caspase-3 and PARP-1were examined.** The relative activities of A20, Bcl-2, Bcl-xL and caspase3 in HCT116 cells treated with ι-car-γ- Fe_2_O_3_ nanocomposite for 24 h were higher than control cells. β-actin was examined as an endogenous control. Significant difference is observed between control and XIAP (p < 0.01), A20 (P < 0.05), Bcl-2 (p < 0.01), Bcl-xL (p < 0.01), caspase3 (p < 0.05) and PARP-1 (p < 0.01). *- p < 0.05, **-p < 0.01.

In order to evaluate the effect of ι-car-γ-Fe_2_O_3_ nanocomposite on the increase in the hypodiploid cell proportion, a cell cycle analysis was performed. Figure 6B shows slight percentage increase in the number of cells in the G2/M phase and decrease in the S-phase with respect to the control. Percentage difference in the G0-phase between the two is not statistically significant. This defective G2/M phase in the nanocomposite treated cells indicate that the entry of the cells into mitosis is checked due to the DNA damage and hence, the cells undergo apoptosis [45]. Cyclin regulatory proteins and p53 pathway may have a significant role in the apoptosis [46]. Nanocomposite is observed to induce accumulation of cells in G1/S phase. Similar results are reported by Haneji et al. for fucoidan-induced cell death [47]. They also reported G1 arrest in human cancer cell, HCT116. ι-car-γ-Fe_2_O_3_ nanocomposite leads to the downregulation of the expression levels of mRNA of XIAP and PARP-1 and upregulation of caspase-3 [48]. Of the members of the IAP protein family, XIAP, has been reported to exert the strongest anti-apoptotic function, as it inhibits caspase-3 indicating that apoptosis is through the mitochondrial pathway [49]. However, Bcl-2, Bcl-xL and caspase-3 are upregulated in nanocomposite treated cells when compared to the control (Figure 7). This indicates that the treatment leads to mitochondrial dysfunction in HCT116 cells. PARP-1 (Poly (ADP-ribose) polymerase 1) is a nuclear enzyme that catalyzes the transfer of ADP-ribose polymers onto itself and other nuclear proteins in response to DNA strand break [50]. It has been widely used as a hallmark of cell apoptosis that play an important role in DNA replication and repair. Downregulation of PARP indicates the incapability of cells to respond to DNA damage and hence induces apopotic cell death [51].

Selective cleavage of 116 kD PARP between Asp214 and Gly215, to generate 89 and 24 kD polypeptides by caspase-3 is a universal phenomenon. This is observed during programmed cell death induced by an apoptotic stimulus [52,53]. However, the Western blot of PARP from nuclear extract of the ι-car-γ-Fe_2_O_3_ nanocomposite treated and control HCT116 cells showed same band density of uncleaved PARP (MW 116 kD) in the treated cells (Additional file 4). No band appeared at 50 kD indicating that the cell death did not involve necrosis [54,55]. The cell death in the treated cells with un-cleavable PARP could be due to the activation of caspase-resistant PARP and the subsequent depletion of intracellular NAD^+^ and ATP [56,53]. Apoptotic cell death due to the augmented levels of TNF-α and Fas were reported in fucoidan treated HL-60 cells [57]. However, it was reported that cells with PARP-null background (PARP^−/−^) exhibited a normal apoptotic response to various stimuli including TNF-α and anti-Fas treatment, suggesting that PARP is dispensable in the apoptotic cascade [58,59]. This could be understood possibly because PARP is not involved in the apoptotic cell death caused by the nanocomposite treated cells.

A20 is not significantly upregulated when compared to the control in ι-car-γ- Fe_2_O_3_ nanocomposite treated cells. It is a nuclear factor-κB (NF-κB) dependent gene that shows both cell-type specific anti-apoptotic or pro-apoptotic functions. Changes in the mRNA expression levels of A20 could be related to both carcinogenesis and inflammatory cell signalling [48]. NF-κB (nuclear factor kappa-light-chain-enhancer of activated B cells) is a eukaryotic transcription factor that contributes equally to cell proliferation or cell death. Tumor necrosis factor-α induced protein 3 (tnfaip3), a gene encoding A20 protein, regulates NF-κB activation by interacting with various components in the upstream signaling pathway. However, both A20 and NF-κB are interrelated, since the former is an NF-κB dependent gene [48]. Recent reports suggest that the expression of A20 is influenced by, tumor development, immune regulation and inflammation [48]. While A20-targeted therapies may certainly add to the chemotherapeutic armamentarium, a better understanding of A20 regulation and its molecular targets and function is highly essential. Similarly, for personalized chemotherapeutic regimen, A20-targeting agents (inducers and inhibitors) for each tumor hold great promise and could be a novel area for research.

Altogether, results confirm that the exposure of HCT116 cells to ι-car-γ-Fe_2_O_3_ nanocomposite resulted in apoptotic cell death, nuclear fragmentation, apoptosome formation, and upregulation of Bcl-2, Bcl-xL and caspase-3 and downregulation of XIAP. It could be speculated that cell death in the nanocomposite treated cells could be due to the mitochondrial ROS and activation of death receptor signaling pathway.

## Methods

### Preparation of ι-car-γ-Fe_2_O_3_ nanocomposite

Maghemite nanoparticles were prepared as described by Russo et al. [60]. These were synthesized by the reduction of ferric chloride (FeCl_3_.6H_2_O) (37 mM) using ammoniacal solution (3.5%) of sodium borohydride (NaBH_4_) (53 mM). The mixture was heated at 100°C for 2 h and kept for overnight aging at room temperature. The aged black product was separated by neodymium magnets (N35, 263–287 KJ/m^3^BH, and 1170–1210 mJ flex density by power magnet). The product was then washed several times with milli-Q water and treated at 400°C for 2 h. Reddish brown particles obtained were dispersed in 3.5 l of milli-Q by ultrasonication for 10 h. This gave a colloidal nanoparticle suspension with good stability.

A known weight (7 mg) of ι-car was dissolved in 10 ml of Milli-Q water at 40°C for 15 min, and 5 ml of γ-Fe_2_O_3_ nanoparticle suspension (4 μg/ml) was added to this drop by drop, under mild stirring. After uniform dispersion by stirring and then sonication for 15 – 30 min, the suspension was lyophilized and stored at 4°C for further study.

### Characterization of ι-car-γ-Fe_2_O_3_ nanocomposite

Size and surface charge of ι-car and ι-car-γ-Fe_2_O_3_ nanocomposite were estimated using Microtrac Particle Analyzer (Zetatrac, India). Surface morphology of the lyophilized products was analyzed using scanning electron microscope (FEI Quanta FEG 200-High Resolution Scanning Electron Microscope). UV spectra of ι-car and ι-car-γ-Fe_2_O_3_ nanocomposites (1 mg/ ml) were obtained using UV/Visible spectrometer (UV/Vis Spectrophotometer, V-550, Jasco Corporation, India; Spectra Manager ver.1.53.01, Jasco). FTIR spectra were recorded using a KBr pellet in FTIR spectrometer (Perkin Elmer, USA). The percentage transmittance (%T) was recorded in the spectral region of 400–4500 cm^−1^ with 20 scan per sample. Powder X-ray diffraction patterns of ι-car and nanocomposite were recorded using CuK α radiation (λ = 0.1541 nm) with Bruker D8 X-ray diffractometer.

### Anticancer activity of ι-car-γ-Fe_2_O_3_ nanocomposite

Human embryonic kidney cell lines (HEK293) and rat myoblast cell lines (L6) were maintained in DMEM and human colon cancer cell line (HCT116) was maintained in RPMI1640, containing 10% FBS and 5% antibiotic in a humidified atmosphere of 5% CO_2_, at 37°C. The cytotoxic activity of ι-car, γ-Fe_2_O_3_ and ι-car-γ-Fe_2_O_3_ nanocomposite were evaluated against these cell lines using 3-[4, 5-dimethythiazol-2-yl]-3, 5-diphenyltetrazolium bromide dye (MTT) [40]. 1 × 10^5^ cells/ ml were seeded in 700 μL of media in the wells of a 24-well microplate and incubated for 24 h. Various concentrations of ι-car (0, 100, 200, 400, 600, 800 and 1000 μg/ml), γ-Fe_2_O_3_ (0, 2.5, 5, 10, 30, 50 and 100 μg/ml) and ι-car-γ-Fe_2_O_3_ nanocomposite (0, 25, 50, 100, 200, 400, 500 μg/ml in DMEM and RPMI 1640, respectively at a pH of 7.4) were added and incubated for 24 h. 70 μl/ well (7.5 mg/ml) of MTT in phosphate buffer saline (PBS) is added to each well and again incubated for 4 h. The medium was removed and 700 μl/well of dimethyl sulfoxide (DMSO) was added to dissolve the formazan. Cell viabilities were determined by measuring the absorbance at 570 nm using a Microplate reader (Enspire, Multimode plate reader, Perkin Elmer, Singapore). Each experiment was repeated thrice. The cell viability (%) was calculated according to the following equation:

$$ \mathrm{Cell}\ \mathrm{viability}\ \left(\%\right)=\left(\mathrm{O}\mathrm{D}57{0}_{\mathrm{sample}}/\mathrm{O}\mathrm{D}57{0}_{\mathrm{control}}\right)\times 100 $$

Where, OD570_sample_ and OD570_control_ represent measurements from the treated and untreated wells, respectively.

### Apoptosis studies

For analysing the morphological changes due to apoptosis, cells were seeded at 3 × 10^5^ cells/ml into the wells of a 6-well plate and cultured for 24 h. Then they were treated with 500 μg/ml of ι-car-γ-Fe_2_O_3_ nanocomposite, washed with PBS and stained with 10 μg/ml of Hoechst 33342 for 30 min at 37°C. The cells were observed using an inverted fluorescent microscope (Leica Microsystems, Germany). The cells were fixed with cold 2% of paraformaldehyde (PFA) for 20 min, washed with cold PBS and stained with 7-aminoactinomycin (7-AAD) for 20 min. They were then observed using an inverted fluorescent microscope (Leica Microsystems, Germany) and photographed. To detect the nuclear damage or chromatin condensation, treated and untreated cells (1 × 10^6^ cells) were harvested using trypsin, washed and mixed with 100 μl of PBS. 10 μl of cells were double-stained using acridine orange (AO) and ethidium bromide (EB) (5 μl of 50 μg/ml). They were then observed and photographed using an inverted fluorescent microscope (Leica Microsystems, Germany). Acridine orange is taken up by both viable and nonviable cells and they emit green fluorescence if intercalated into double-stranded nucleic acid (DNA) or red fluorescence if bound to single stranded nucleic acid (RNA). Ethidium bromide is taken up only by nonviable cells and so emits red fluorescence by intercalation into DNA. Based on the fluorescence emission and the morphological aspect of chromatin condensation in the stained nuclei, cells are classified as viable cells (uniform bright green nuclei with an organized structure), apoptotic cells (have intact membrane but have started to undergo DNA cleavage, so have green nuclei but perinuclear chromatin condensation is visible as bright green patches or fragments), late apoptotic cells (orange to red nuclei with condensed or fragmented chromatin) and necrotic cells (uniformly orange to red nuclei with a condensed structure). The study was done in triplicates. Percentage of apoptotic and necrotic cells were calculated using the following formulae,

$$ \begin{array}{l}\%\ \mathrm{A}\mathrm{poptotic}\ \mathrm{cells}=\left(\mathrm{V}\mathrm{A}+\mathrm{N}\mathrm{V}\mathrm{A}\right)/\left(\mathrm{V}\mathrm{N}+\mathrm{V}\mathrm{A}+\mathrm{N}\mathrm{V}\mathrm{N}+\mathrm{N}\mathrm{V}\mathrm{A}\right)\times 100\hfill \\ {}\%\ \mathrm{N}\mathrm{ecrotic}\ \mathrm{cells}=\left(\mathrm{N}\mathrm{V}\mathrm{N}\right)/\mathrm{V}\mathrm{N}+\mathrm{V}\mathrm{A}+\mathrm{N}\mathrm{V}\mathrm{N}+\mathrm{N}\mathrm{V}\mathrm{A}\times 100\hfill \end{array} $$

Where,

- VN = viable cells with normal nuclei (bright green chromatin with organized structure),
- VA = viable cells with apoptotic nuclei (bright green chromatin which is highly condensed or fragmented)
- NVN = nonviable cells with normal nuclei (bright orange chromatin with organized structure),
- NVA = nonviable cells with apoptotic nuclei (bright orange chromatin which is highly condensed or fragmented).

The morphology of apoptotic cells was determined with the help of an annexin V-FITC and PI double-staining technique [41]. HCT116 cells were seeded onto 6-well plates (5 × 10^3^ cells/well) and cultured for 24 h. After treatment with or without ι-car-γ-Fe_2_O_3_ for 24 h, they were stained with the annexin V-FITC labeling solution and 5 μl of PI (50 μg/ml). The plates were incubated for 15 min in the dark, and then images of the cells were acquired using BD FACSVerse™ flow cytometer. The nucleus of the cells with apoptotic morphology (condensation/fragmentation) or annexin V-positive cells was analyzed using the BD FACSuite™ software (BD Biosciences, Germany). For each analysis, 3000 cells were recorded.

Distribution of the cells in various phases in the cycle was determined using a flow cytometre [41]. After treatment with or without ι-car-γ-Fe_2_O_3_, the cells were harvested using trypsin, washed with cold PBS and incubated with 10 μg/ml of RNase A for 30 min at room temperature. PI (5 μl of 5 μg/ml) was added to the cell suspension and they were incubated for 10 min in the dark. The DNA content was analyzed by flow cytometre (BD FACSVerse™ flow cytometer, BD FACSuite™ software, BD Biosciences, Germany). The proportion of cells in G1, S and G2/ M phases were determined. 10000 cells were recorded during each reading.

ROS plays a key role in the oxidative stress and its imbalance causes cellular damage. To quantify ROS, cells were incubated with ι-car-γ-Fe_2_O_3_ and labeled with 2 μl of 20 mM stock solution of 2′, 7′-dichlorofluorescin diacetate (DCFH-DA) at 37°C for 30 min. The cellular fluorescence intensity was measured after washing the cells with PBS at an excitation and emission wavelengths of 485 and 530 nm, respectively, using a Microplate reader (Enspire, Multimode plate reader, Perkin Elmer, Singapore). DCFH-DA enters the cell where it reacts with ROS to form the highly fluorescent dichlorofluorescein (DCF) [61].

### Real-time polymerase chain reaction

Cells were harvested after 24 h of treatment with PBS and 500 μg/ml of ι-car-γ-Fe_2_O_3_. Total-RNA was extracted using the RNAiso Plus (Total RNA extraction reagent, Takara Bio Inc., Japan) [62]. The quality of RNA was evaluated by measuring the absorbance (Nanodrop 2000 Spectrophotometer, Thermoscientific, USA) at 260 and 280 nm which indicates its concentration and purity. The High Capacity cDNA Reverse Transcription Kits Protocol (Life Technologies, India) was used to prepare the cDNA according to the manufacturer’s instructions. All the samples were stored at −20°C. Quantitative PCR was conducted in 20 μl reactions containing KAPA SYBR® FAST qPCR kit (KAPA Biosystems, Wilmington, Massachusetts) using the Mastercycler ep realplex^4^ PCR system (Eppendorf, Australia). The primers are shown in Table 3. Reaction mixtures were incubated for an initial denaturation at 95°C for 3 min followed by 40 cycles of 95°C for 3 sec, 56°C for 15 sec and 72°C for 15 sec. For each sample, the expression level of each mRNA was quantified as the cycle threshold difference (ΔΔC_t_) with respect to β-actin as internal housekeeping gene. Real time PCR data were analyzed using the 2^-ΔΔCt^ relative quantification method using the given formulae. All the reactions were performed in triplicate.

$$ \varDelta \varDelta {\mathrm{C}}_{\mathrm{t}}=\left({\mathrm{C}}_{\mathrm{t}}\left(\mathrm{target},\ \mathrm{untreated}\right)-{\mathrm{C}}_{\mathrm{t}}\left(\mathrm{r}\mathrm{e}\mathrm{f},\ \mathrm{untreated}\right)\right)-\left({\mathrm{C}}_{\mathrm{t}}\left(\mathrm{target},\ \mathrm{treated}\right)-{\mathrm{C}}_{\mathrm{t}}\left(\mathrm{r}\mathrm{e}\mathrm{f},\ \mathrm{treated}\right)\right) $$

where

- C_t_ (target, untreated) = C_t_ value of gene of interest in untreated sample
- C_t_ (ref, untreated) = C_t_ value of control gene in untreated sample
- C_t_ (target, treated) = C_t_ value of gene of interest in treated sample
- C_t_ (ref, treated) = C_t_ value of control gene in treated sample

**Table 3:** **Primers for real-time PCR**

	**Forward:**	**Reverse:**
β-actin	5′-CTCACCATGGATGATGATATCGC	5′-AGGAATCCTTCTGACCCATGC
XIAP	5′- GCGCGAAAAGGTGGACAAGT	5′- CTGCTCGTGCCAGTGTTGAT
A20	5′-AGTCTGCAGTCTTCGTGGC	5′-AGTCCTGGTCAAGGCAGGAG
Bcl-2	5′-TCCTGGCTGTCTCTGAAGACT	5′-AGCCTGCAGCTTTGTTTCAT
Bcl-xL	5′- ACTCTTCCGGGATGGGGTAA	5′- AATGAGGTGCAAAGTCCCCC
PARP-1	5′-CTACTCGGTCCAAGATCGCC	5′-TGAAAAAGCCCTAAAGGCTCA

### Western blot analysis

Untreated HCT 116 (control) and cells treated with 500 μg/ml of ι-car-γ-Fe_2_O_3_ nanocomposite for 24 h, were lysed and whole cell lysates were extracted with RIPA (Radio-Immunoprecipitation Assay) lysis buffer and protein concentrations were measured using Nanodrop 2000 Spectrophotometer (Thermoscientific, USA). Samples containing equal concentrations of protein were separated by 10% SDS-PAGE (Bio-Rad Mini Protean Tetra Cell, India) and transferred to nitrocellulose membranes and blot device (Kiran X-ray Cassette, Kiran Medical Systems Ltd, India). The nitrocellulose sheet was incubated for 1 h with 5% milk (v/v). Pre-stained and un-stained broad range protein ladder (Puregene, Genetix Biotech Asia Pvt. Ltd., India) was used to distinguish MW of PARP and its cleavage products, noting that the MW of PARP was 116 kD, the apoptotic degradation product was 89 kD, and the necrotic products were 50 kD [54]. The membranes were blocked with 5% skimmed milk in phosphate saline buffer with Tween for 1 h at room temperature and maintained overnight at 4°C with PARP antibody (rabbit) prepared in skimmed milk (Cell Signaling Technology Inc., India). Membranes were then incubated with anti-rabbit horseradish peroxidase-conjugated secondary antibodies (Cell Signaling Technology Inc., India) at room temperature for 1 h 30 min. Antibody-bound proteins were detected using enhanced chemiluminescence (ECL).

### Statistical analysis

Groups of data were compared with Analysis of variance (ANOVA) using SPSS software (Chicago, IL, USA). A p value less than 0.05 was considered to be statistically significant.

## Conclusions

This novel, biocompatible and biodegradable hybrid nano ι-CGN-γ-Fe_2_O_3_ composite has been successfully prepared and characterized by various analytical techniques such as UV-spectroscope, HR-SEM, DLS, FTIR and Powder XRD. The results confirm the integration of maghemite nanoparticles to the sulphate groups of carrageenan. They have properties that can make them attractive and lucrative in biomedical applications. Tuning the surface properties of this nanocomposite by changing the concentration ratio of ι-car and γ-Fe_2_O_3_ can make them more dynamic in targeting cancer cells. Initiating the inhibition of growth in cancer cells without being cytotoxic to normal cell lines makes it a promising nanovector in drug delivery. The nanocomposite induces alterations in the cancer cells that are related to the apoptosis process through ROS-mediated mitochondrial pathway, by increasing ROS production and inducing mitochondrial oxidative damage, combined with upregulation of caspase3 and activation of death receptor signaling pathway. This is also accompanied by upregulation of Bcl-2 and Bcl-xL indicating the damage of mitochondrial wall and probable production of pro-apoptotic proteins. Hence the ι-car-γ-Fe_2_O_3_ nanocomposite has potential as an efficient chemotherapeutic agent, since targeting of chemotherapeutic agents is related to its capacity to induce apoptosis.

**Figure 8:**
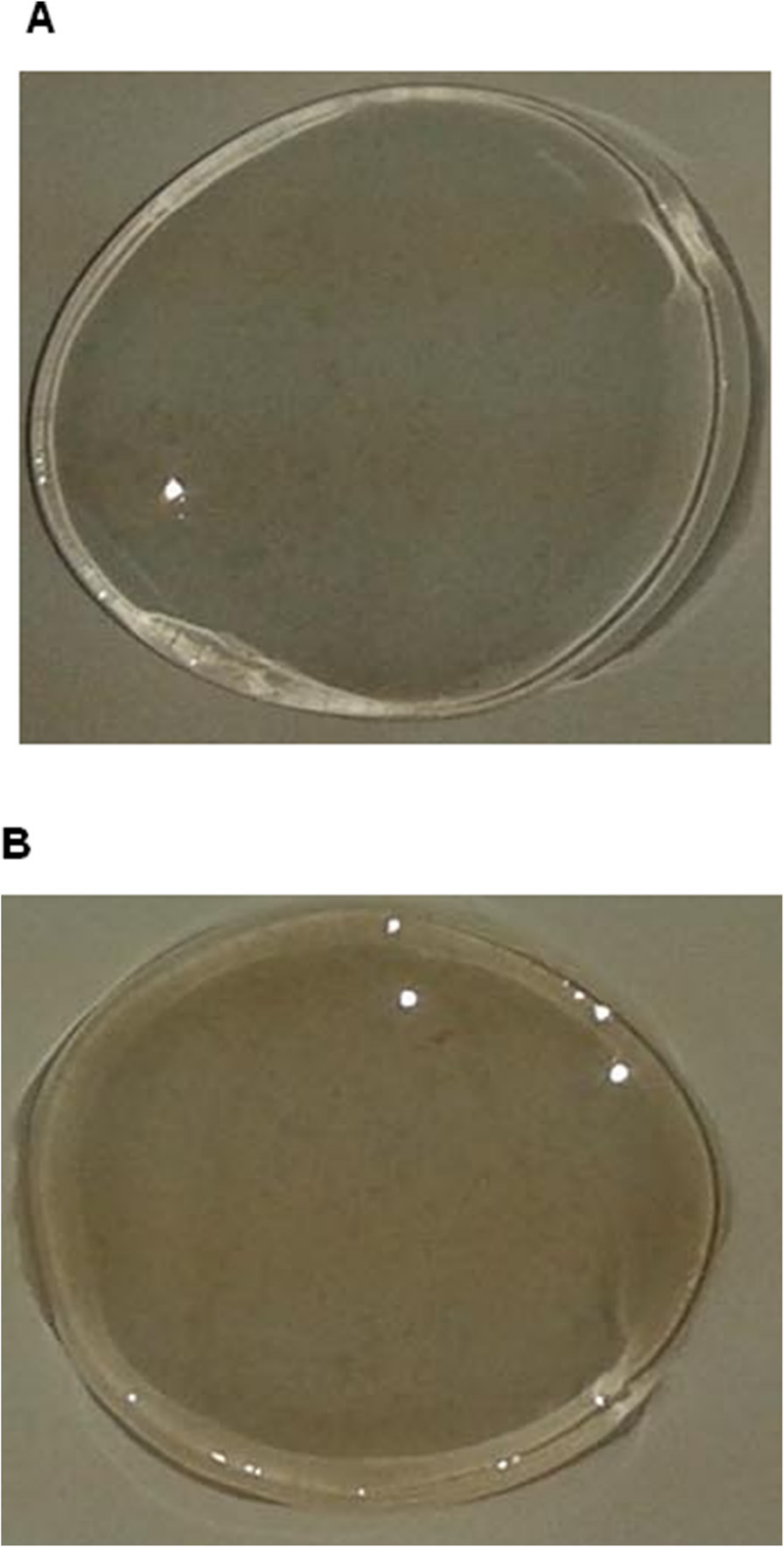
**Gel Formation: Gels from (A)** **ι** **-car with cations and (B)** **ι** **-car with** **γ** **-Fe**_**2**_ **O**_**3**_ **.**

Our studies on gelation (data not shown, except Figure 8) showed that ι-CGN with γ-Fe_2_O_3_ nanoparticles did not require any addition of cations, as γ-Fe_2_O_3_ nanoparticles themselves served as cations in neutralizing the charges and in promoting gelation. Gel forming ability of the nanocomposite can be exploited in targeted drug delivery and cancer hyperthermia. Surface charge, gel forming ability and the magnetic properties of our ι-CGN-γ-Fe_2_O_3_ composite can be tuned to fit into appropriate applications by changing the nanoparticle concentration. Research to explore their significance in chemotherapy, controlled drug delivery and hyperthermia are to be continued.

## Additional files

Additional file 1:
**Particle size distribution of ι-car-γ-Fe**_**2**_ **O**_**3**_ **nanocomposite.**
Additional file 2:
**UV spectrum of (A) ι-car (B) γ-Fe**_**2**_ **O**_**3**_ **nanoparticles (C) ι-car-γ-Fe**_**2**_ **O**_**3**_ **nanocomposite.**
Additional file 3:
**Cell Proliferation Assay: Viability of HEK293, L6 and HCT116 cells treated with (A) ι-car and (B) γ-Fe**_**2**_ **O**_**3**_ **.** Significant difference (*, p < 0.01) is observed in HCT116 treated with ι-car. γ-Fe_2_O_3_ showed significant decrease in cell viability in the cell lines with increased concentration. Additional file 4:
**Western Blot analysis of poly (ADP-ribose) polymerase (PARP) in cell lysate obtained from control and ι-car-γ-Fe**_**2**_ **O**_**3**_ **nanocomposite treated HCT116 cells after 24 hr of treatment.** Shown are the full-length PARP (116 kD). PARP cleavage in nanocomposite treated cells were not observed.

## Abbreviations

MRI: Magnetic Resonance Imaging
γ- Fe_2_O_3_: Gamma maghemite
ι *–*car: Iota *–*Carrageenan
G4S: D-galactose-4-sulphate
DA2S: 3, 6-anhydro-D-galactose-2-sulfate
HEK293: Human embryonic kidney cell lines
L6: Rat myoblast cell lines
HCT116: Human colon cancer cell line
DMEM: Dulbecco’s modified Eagle’s medium
RPMI: Roswell Park Memorial Institute medium
FBS: Fetal bovine serum
MTT: 3-(4, 5-dimethylthiazol-2-yl)-2, 5-diphenyltetrazolium bromide
7-AAD: 7-Aminoactinomycin
