# Supplementary figures and images for: Biocompatible ι-carrageenan-γ-maghemite nanocomposite for biomedical applications – synthesis, characterization and in vitro anticancer efficacy
Source: J Nanobiotechnology. 2015 Mar 3;13:18. doi: 10.1186/s12951-015-0079-3 (PMC4356133; doi:10.1186/s12951-015-0079-3)

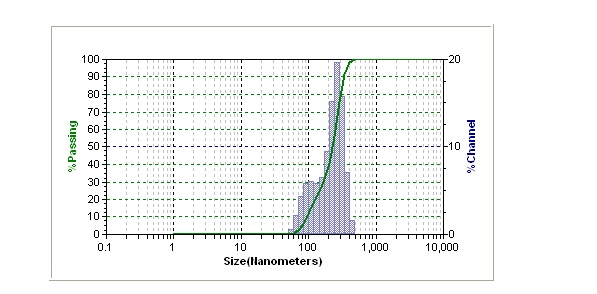

Supplement: Additional file 1: — Particle size distribution of ι-car-γ-Fe 2 O 3 nanocomposite. [file 12951_2015_79_MOESM1_ESM.png]

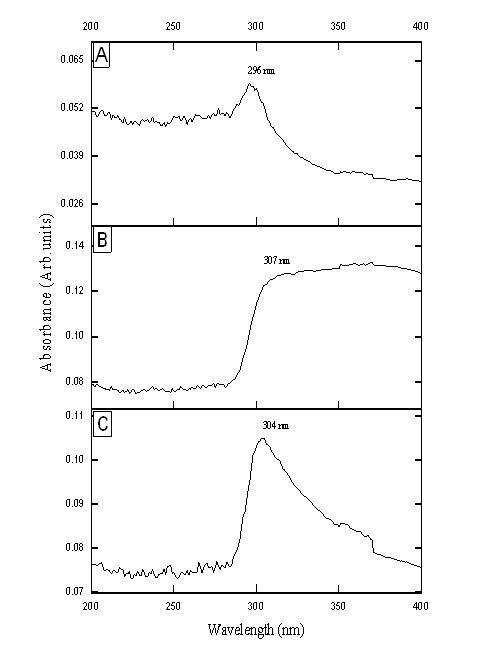

Supplement: Additional file 2: — UV spectrum of (A) ι-car (B) γ-Fe 2 O 3 nanoparticles (C) ι-car-γ-Fe 2 O 3 nanocomposite. [file 12951_2015_79_MOESM2_ESM.png]

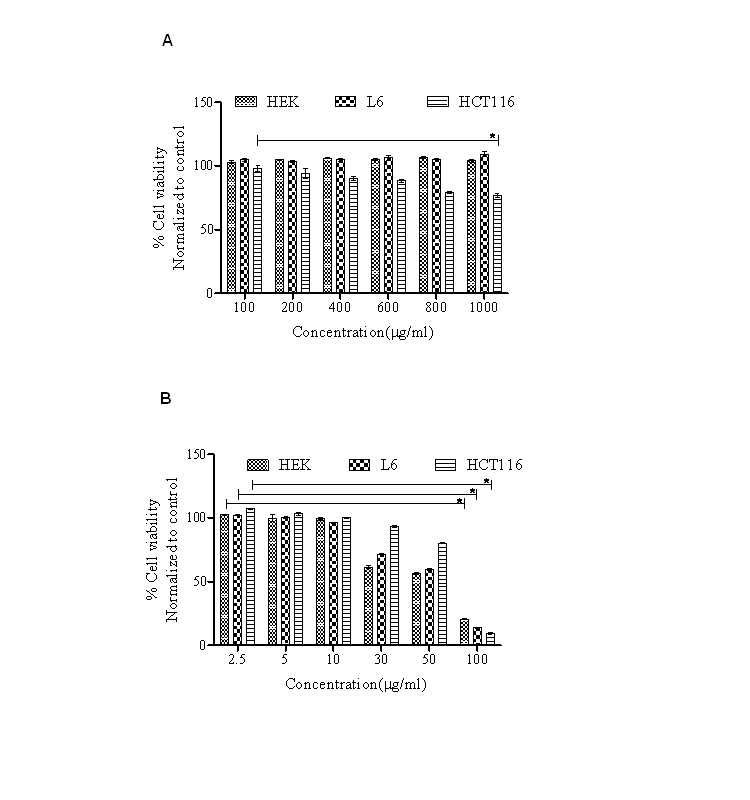

Supplement: Additional file 3: — Cell Proliferation Assay: Viability of HEK293, L6 and HCT116 cells treated with (A) ι-car and (B) γ-Fe 2 O 3 . Significant difference (*, p < 0.01) is observed in HCT116 treated with ι-car. γ-Fe2O3 showed significant decrease in cell viability in the cell lines with increased concentration. [file 12951_2015_79_MOESM3_ESM.png]

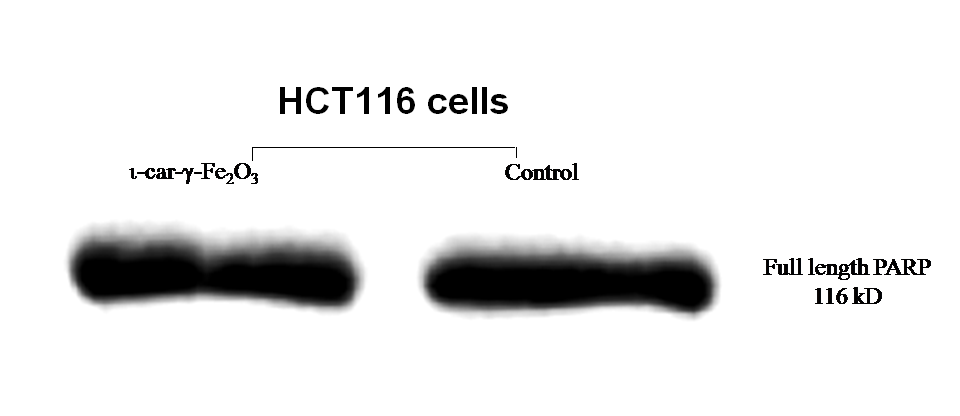

Supplement: Additional file 4: — Western Blot analysis of poly (ADP-ribose) polymerase (PARP) in cell lysate obtained from control and ι-car-γ-Fe 2 O 3 nanocomposite treated HCT116 cells after 24 hr of treatment. Shown are the full-length PARP (116 kD). PARP cleavage in nanocomposite treated cells were not observed. [file 12951_2015_79_MOESM4_ESM.png]
